# Sustained Akt signaling in articular chondrocytes causes osteoarthritis via oxidative stress-induced senescence in mice

**DOI:** 10.1038/s41413-019-0062-y

**Published:** 2019-08-05

**Authors:** Jing Xie, Jingting Lin, Min Wei, Yan Teng, Qi He, Guan Yang, Xiao Yang

**Affiliations:** 1 State Key Laboratory of Proteomics, Beijing Proteome Research Center, National Center for Protein Sciences (Beijing), Beijing Institute of Lifeomics, Beijing, 102206 China; 2 Department of Orthopaedics, Chinese PLA General Hospital, Beijing, 100853 China

**Keywords:** Physiology, Bone

## Abstract

Osteoarthritis (OA) is an age-related disorder that is strongly associated with chondrocyte senescence. The causal link between disruptive PTEN/Akt signaling and chondrocyte senescence and the underlying mechanism are unclear. In this study, we found activated Akt signaling in human OA cartilage as well as in a mouse OA model with surgical destabilization of the medial meniscus. Genetic mouse models mimicking sustained Akt signaling in articular chondrocytes via *PTEN* deficiency driven by either *Col2a1-Cre* or *Col2a1-Cre*^*ERT2*^ developed OA, whereas restriction of Akt signaling reversed the OA phenotypes in *PTEN*-deficient mice. Mechanistically, prolonged activation of Akt signaling caused an accumulation of reactive oxygen species and triggered chondrocyte senescence as well as a senescence-associated secretory phenotype, whereas chronic administration of the antioxidant N-acetylcysteine suppressed chondrocyte senescence and mitigated OA progression in *PTEN*-deficient mice. Therefore, inhibition of Akt signaling by PTEN is required for the maintenance of articular cartilage. Disrupted Akt signaling in articular chondrocytes triggers oxidative stress-induced chondrocyte senescence and causes OA.

## Introduction

Osteoarthritis (OA) is an age-related disease characterized by articular cartilage degeneration, which is the leading cause of pain and disability worldwide.^1^ The serine/threonine kinase Akt is activated by extracellular signals that activate phosphatidylinositol 3-kinase (PI3K), while the phospholipid phosphatase PTEN negatively regulates the activity of Akt by negating the activity of PI3K.^2^ PTEN-modulated PI3K/Akt signaling has been widely studied for its central role in physiology and disease, particularly in cancer, where it has become an attractive pharmacological target.^3^ Extensive studies have revealed the function of the PTEN/Akt pathway in chondrocytes during endochondral ossification. Deletion of *Akt1* results in delayed calcification,^4–6^ while Akt activation in embryonic chondrocytes promotes chondrocyte proliferation and inhibits hypertrophic differentiation.^7–9^ However, the in vivo function of Akt signaling in the maintenance of articular cartilage homeostasis and in OA development is largely undefined. Many studies based on in vitro experiments have reported contradictory results regarding the function of Akt signaling in cartilage homeostasis. PI3K/Akt signaling has been shown to play a chondroprotective role by regulating chondrocyte survival, proliferation and extracellular matrix synthesis.^10–12^ In contrast, some studies have reported a detrimental effect of the PI3K/Akt pathway on OA, which might be achieved through transduction of procatabolic stimuli or inhibition of articular chondrocyte autophagy.^13,14^ Nevertheless, the causal link between disruptive PTEN/Akt signaling in articular chondrocytes and OA pathogenesis is unclear.

Aging is appreciated as a major risk factor for the prevalence of OA.^15^ Although senescent chondrocytes are usually found in OA cartilage tissue, the causative role of senescent cells in OA pathogenesis was not established in vivo until recently.^16^ However, little is known about the mechanisms that modulate the senescence of articular chondrocytes. Excessive reactive oxygen species (ROS) have been demonstrated to be inducers of cellular senescence.^17^ In OA chondrocytes, ROS-induced oxidative damage has been implicated as the major cause of cellular senescence, as indicated by reduced capacities for chondrocyte proliferation, survival and extracellular matrix (ECM) synthesis, as well as increased production of matrix-degrading enzymes.^18^ Insulin/IGF-1/PI3K/Akt/forkhead-box O (FOXO) signaling is a major pathway that regulates ROS generation and cell senescence.^19,20^ FoxO transcriptional factors play a chondroprotective role by supporting oxidative stress resistance.^21–23^ However, it remains unknown whether Akt signaling serves as a chondroprotective pathway or executes a prosenescence response during OA pathogenesis in vivo.

In the current study, we employed a series of mouse models to investigate the function and relevant mechanisms of Akt signaling in OA development. We provide in vivo genetic evidence that inhibition of Akt signaling by PTEN is required for the maintenance of articular cartilage, which is achieved through inhibition of oxidative stress-induced chondrocyte senescence. These findings will provide new insights into the physiopathologic mechanisms and the relevant pharmacological targets of human OA.

## Results

### Akt signaling is activated in human OA and experimental OA mouse models

**Fig. 1:**
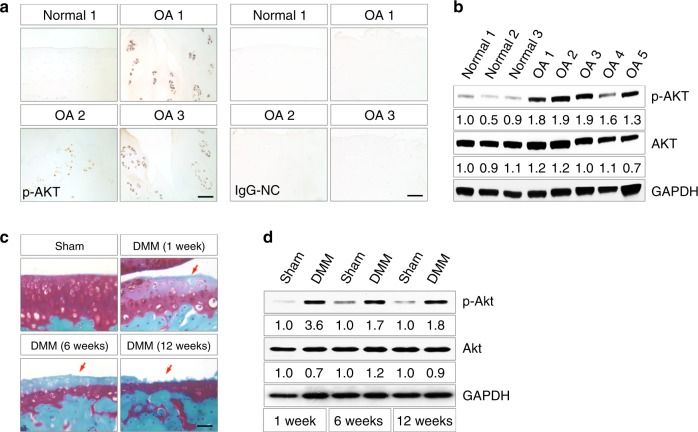
Akt signaling is activated during OA development. **a** Representative images of immunohistochemical staining of p-AKT in human articular cartilage from traumatic knee joints (normal) and knee arthroplasties of OA patients (OA). Nonimmunized rabbit IgG was used as the negative control (IgG-NC). **b** Western blot analysis for p-AKT levels in human normal cartilage and OA cartilage. Quantitative densitometry results are shown below. The GAPDH protein serves as an endogenous normalizer, and the results of each band are then normalized to the value of the “Normal 1” sample. **c** Representative images of safranin O staining of mouse knee joints at 1, 6, and 12 weeks after Sham or DMM surgery performed at 8-week-old (*n* = 3 per group). Arrows denote the articular surface of knee joints that show progressive loss of integrity. **d** Western blot analysis for p-Akt levels in articular cartilage of knee joints at 1, 6, and 12 weeks after Sham or DMM surgery. Quantitative densitometry results are shown below. The GAPDH protein serves as an endogenous normalizer. Scale bars: 100 µm in (**a**); 40 µm in (**c**)

We first examined the level of phosphorylated AKT (p-AKT) in human OA cartilage. In articular cartilages from traumatic knee joints, little p-AKT immunohistochemical staining was observed, while in those from knee arthroplasties of OA patients, notable p-AKT staining was present within the clustered articular chondrocytes (Fig. 1a). Western blot analyses confirmed the significantly increased p-AKT level in articular cartilage from OA patients (Fig. 1b), indicating that Akt signaling is activated in human OA chondrocytes.

We then employed a surgical mouse model involving destabilization of the medial meniscus (DMM) to examine the status of Akt signaling during OA development.^24^ The histological severity of experimental OA at different stages was demonstrated by safranin O staining (Fig. 1c). The level of p-Akt significantly increased and was sustained with the progression of experimental OA in mice (Fig. 1d). These data demonstrate that Akt signaling is activated during OA pathogenesis.

### Sustained Akt signaling in articular chondrocytes causes OA in mice

**Fig. 2:**
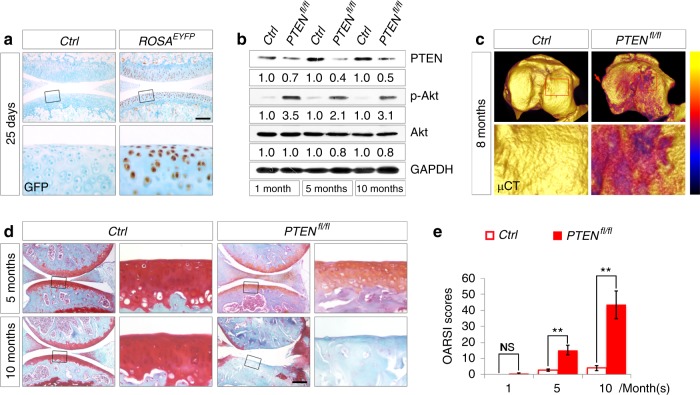
*PTEN* deficiency in articular chondrocytes causes OA in mice. **a** Representative images of immunohistochemical staining of EYFP in sagittal sections of 25-day-old *Ctrl* and *ROSA*^*EYFP*^ knee joints (*n* = 2 per group). The framed area in each picture is shown below at a higher magnification. **b** Western blot analyses for the levels of PTEN, Akt, and p-Akt in articular cartilage from *Ctrl* and *PTEN*^*fl/fl*^ mice at 1, 5, and 10 months. Quantitative densitometry results are shown below. The GAPDH protein serves as an endogenous normalizer. **c** Representative color-coded 3D-reconstructed μCT images of the tibial plateau from *Ctrl* and *PTEN*^*fl/fl*^ mice at 8 months (*n* = 3 per group). A color scale bar ranging from yellow to black is shown to indicate respective HU values from low to high. The arrow denotes an osteophyte at the medial tibial plateau. The framed area in each picture is shown below at a higher magnification. **d** Representative images of safranin O staining of *Ctrl* and *PTEN*^*fl/fl*^ knee joints at 5 and 10 months (*n* = 8 per group). The framed area in each picture is shown on the right at a higher magnification. **e** Quantified pathological changes of *Ctrl* and *PTEN*^*fl/fl*^ knee joints at 1, 5, and 10 months. Each value represents the mean ± SEM (*n* = 8 per group). NS: not significant; **P *<* 0.01. Scale bars: 200 µm in (**a**); 250 µm in (**d**)

To determine the contribution of sustained activation of Akt signaling to OA development, we employed a conditional gene knockout mouse (*Col2a1-Cre*;*PTEN*^*fl/fl*^, further referred to as *PTEN*^*fl/fl*^) in which the *PTEN* gene was specifically inactivated in chondrocytes by the *Col2a1-Cre* transgene.^8^ *Col2a1-Cre* activity in articular chondrocytes was confirmed by the expression of EYFP in *Col2a1-Cre*;*ROSA*^*EYFP*^ reporter mice (*ROSA*^*EYFP*^) (Fig. 2a). Western blot analyses showed decreased levels of PTEN and increased levels of p-Akt in articular cartilage from *PTEN*^*fl/fl*^ mice compared to that from control littermates (*Col2a1-Cre*;*PTEN*^*fl/+*^ or *Col2a1-Cre*, further referred to as *Ctrl*) at 1, 5 and 10 months of age (Fig. 2b). Reconstructed 3D μCT images of the 8-month-old tibial plateau showed evident roughness and erosion throughout the articular surface of *PTEN*^*fl/fl*^ mice, in sharp contrast to that of the *Ctrl* mice (Fig. 2c). Histological observation demonstrated evident OA phenotypes in 5-month-old *PTEN*^*fl/fl*^ mice. The thickness and organization of articular cartilage deteriorated, in addition to reduced staining for proteoglycan (Fig. 2d). Severe depletion of articular cartilage was observed in 100% of 10-month-old *PTEN*^*fl/fl*^ mice (Fig. 2d). OA Research Society International (OARSI) histopathologic scores, including subchondral bone changes, quantified the dramatically increased lesions in *PTEN*^*fl/fl*^ articular cartilage as the mice grew older (Fig. 2e). The histopathologic grading of synovial changes, subchondral bone thickening and osteophyte formation also demonstrated the lesions in *PTEN*^*fl/fl*^ mice (Supplementary Figs. S1–S3).

**Fig. 3:**
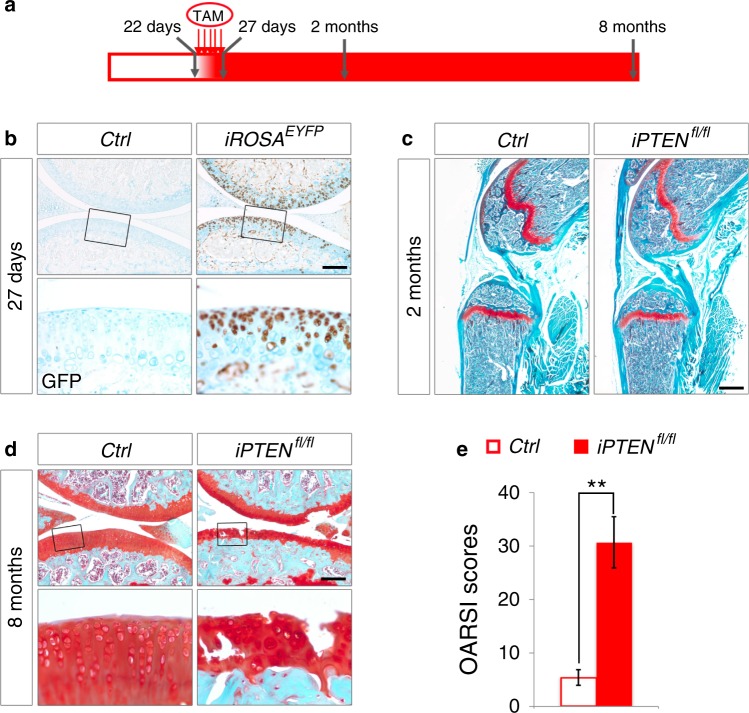
Induced *PTEN* deficiency in adult articular chondrocytes causes OA phenotypes in mice. **a** Schematic diagram showing the protocol of tamoxifen administration for starting *ROSA*^*EYFP*^ expression or ablating the *PTEN* gene in articular chondrocytes. Five successive doses of tamoxifen were injected every day since 22-day-old. Knee joints were analyzed at 27 days, 2 months and 8 months of age. **b** Representative images of immunohistochemical staining of EYFP in articular cartilage from 27-day-old *Ctrl* and *iROSA*^*EYFP*^ mice (*n* = 3 per group). Tamoxifen was injected since 22-day-old. The framed area in each picture is shown below at a higher magnification. **c** Representative images of safranin O staining of hind limbs from 2-month-old *Ctrl* and *iPTEN*^*fl/fl*^ mice (*n* = 4 per group). Tamoxifen was injected since 22-day-old. **d** Representative images of safranin O staining of knee joints from 8-month-old *Ctrl* and *iPTEN*^*fl/fl*^ mice (*n* = 12 per group). Tamoxifen was injected since 22-day-old. The framed area in each picture is shown below at a higher magnification. **e** Quantified pathological changes of each group at 8 months. Each value represents the mean ± SEM (*n* = 12 per group). **P *<* 0.01. Scale bars: 250 µm in (**b**) and (**d**), 800 µm in (**c**)

We have previously reported dyschondroplasia resulting from *PTEN* deficiency in the growth plate of *PTEN*^*fl/fl*^ mice.^8^ Although dyschondroplasia does not occur in articular cartilage, we employed inducible *Col2a1-Cre*^*ERT2*^ transgenic mice to exclude the possible influences of growth plate deformities in young *PTEN*^*fl/fl*^ mice,^25^ and we assessed OA development in *Col2a1-Cre*^*ERT2*^*; PTEN*^*fl/fl*^ mutant mice (further referred to as *iPTEN*^*fl/fl*^ mice). Five successive doses of tamoxifen injection (Fig. 3a) induced Cre activity in most of the articular chondrocytes of *Col2a1-Cre*^*ERT*^; *ROSA*^*EYFP*^ reporter mice (*iROSA*^*EYFP*^) (Fig. 3b). Activation of Akt signaling beyond the key stages of endochondral bone development did not cause visible skeletal defects in 2-month-old *iPTEN*^*fl/fl*^ mice (Fig. 3c) but resulted in evident OA phenotypes at 8 months of age (Fig. 3d, e).

**Fig. 4:**
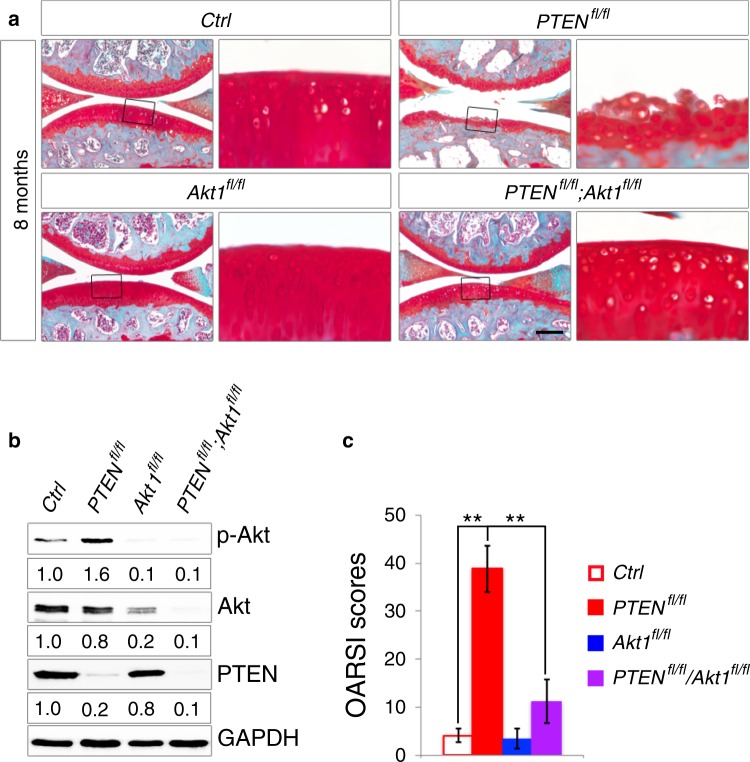
Genetic inhibition of *Akt1* rescues OA development in *PTEN*-deficient mice. **a** Representative images of safranin O staining of knee joints from each genotype at 8 months (*n* = 10 per group). The framed area in each picture is shown on the right at a higher magnification. **b** Western blot analyses for the levels of p-Akt, Akt and PTEN in articular cartilage from each genotype. Quantitative densitometry results are shown below. The GAPDH protein serves as an endogenous normalizer. **c** Quantified pathological changes in knee joints from each genotype at 8 months of age. Each value represents the mean ± SEM (*n* = 10 per group). **P *<* 0.01. Scale bar: 250 µm

We next examined whether OA development in *PTEN*-deficient mice was primarily caused by Akt activation. We reduced Akt expression in chondrocytes by breeding *Col2a1-Cre*; *PTEN*^*fl/fl*^ mice with *Akt1*^*fl/fl*^ mice.^26^ *Akt1* deletion significantly reduced the total cellular level of Akt protein in articular chondrocytes of *Col2a1-Cre*; *PTEN*^*fl/fl*^*;Akt1*^*fl/fl*^ (further referred to as *PTEN*^*fl/fl*^*;Akt1*^*fl/fl*^) mice (Fig. 4b), indicating that Akt1 was the most highly expressed isoform in articular chondrocytes. In contrast to the systemic *Akt1*^−*/*−^ mice,^5^ conditional *Col2a1-Cre*; *Akt1*^*fl/fl*^ mice appeared normal and did not show visible defects in skeletal development (Supplementary Fig. S4). Interestingly, all *PTEN*^*fl/fl*^;*Akt1*^*fl/fl*^ compound-mutant mice displayed a dramatic improvement in articular cartilage integrity, as evidenced by histological examination (Fig. 4a) and OA scores (Fig. 4c). These results demonstrate that *PTEN* deficiency in articular chondrocytes initiates OA development in mice through sustained activation of Akt signaling.

### Sustained PI3K/Akt signaling causes oxidative stress-related senescence in articular chondrocytes

**Fig. 5:**
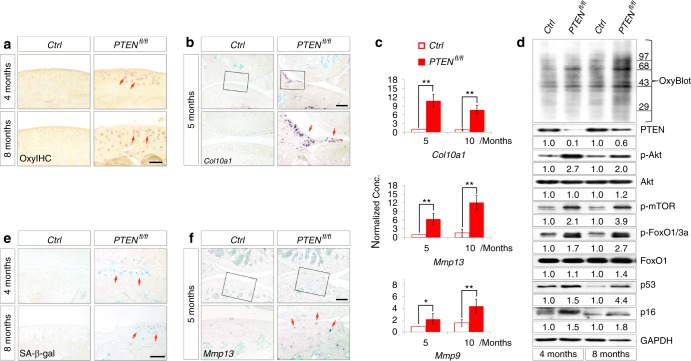
Sustained Akt signaling in articular chondrocytes causes oxidative stress-related senescence. **a** Representative images of OxyIHC staining of articular cartilage from *Ctrl* and *PTEN*^*fl/fl*^ mice at 4 and 8 months (*n* = 4 per group). Red arrows denote the positive staining within articular chondrocytes of *PTEN*^*fl/fl*^ mice. **b** Representative images of *Col10a1* in situ hybridization analyses of knee joints from *Ctrl* and *PTEN*^*fl/fl*^ mice at 5 months (*n* = 3 per group). The framed area in each picture is shown below at a higher magnification. Red arrows denote the positively stained cells within the articular cartilage and meniscus of *PTEN*^*fl/fl*^ mice. **c** Representative real-time PCR analyses of *Col10a1*, *Mmp13*, and *Mmp9* expression in articular cartilage from *Ctrl* and *PTEN*^*fl/fl*^ mice at 5 and 10 months. Each value represents the mean ± SEM (*n* = 3 per group). *P *<* 0.05; **P *<* 0.01. **d** OxyBlot analysis for protein oxidation and western blot analyses for the levels of molecules involved in PTEN/Akt senescence axis. Samples were collected from articular cartilage of *Ctrl* and *PTEN*^*fl/fl*^ mice at 4 and 8 months. Quantitative densitometry results are shown below. The GAPDH protein serves as an endogenous normalizer. **e** Representative images of SA-β-gal staining of articular cartilage from *Ctrl* and *PTEN*^*fl/fl*^ mice at 4 and 8 months (*n* = 4 per group). Red arrows denote the senescent articular chondrocytes in *PTEN*^*fl/fl*^ mice. **f** Representative images of *Mmp13* in situ hybridization analysis of knee joints from *Ctrl* and *PTEN*^*fl/fl*^ mice at 5 months (*n* = 3 per group). The framed area in each picture is shown below at a higher magnification. Red arrows denote the positively stained cells within the articular cartilage of *PTEN*^*fl/fl*^ mice. Scale bars: 50 µm in (**a**) and (**e**), 250 µm in (**b**) and (**f**)

The age-related progression of OA phenotypes in *Col2a1-Cre*; *PTEN*^*fl/fl*^ mice indicates that chondrocyte senescence might be accelerated by prolonged PI3K/Akt signaling. Oxidative stress induced by ROS is the major cause of chondrocyte senescence.^27^ We, therefore, employed OxyIHC to detect protein oxidation in chondrocytes and found that oxidative stress was significantly increased in articular chondrocytes from 4- and 8-month-old *PTEN*^*fl/fl*^ mice (Fig. 5a, red arrows). The hypertrophy of chondrocytes is also indicative of intracellular ROS.^28,29^ In 5-month-old *PTEN*^*fl/fl*^ mice, the expression of *Col10a1*, a well-established marker for hypertrophic differentiation of chondrocytes, manifested within the articular cartilage and meniscus (Fig. 5b, red arrows). Elevated expression of *Col10a1* mRNA in cartilage from 5- and 10-month-old *PTEN*^*fl/fl*^ mice was confirmed by real-time PCR (Fig. 5c). OxyBlot is a western blot method that quantifies protein oxidation in tissues based on a principle identical to that of OxyIHC. As shown by OxyBlot, *PTEN* deficiency led to elevated intracellular oxidative stress in articular chondrocytes from 4- and 8-month-old *PTEN*^*fl/fl*^ mice, which was accompanied by increased levels of p-Akt, p-mTOR, and p-FoxO1/3a (Fig. 5d). Consistently, deletion of *PTEN* triggered senescence of articular chondrocytes, as indicated by SA-β-gal staining within articular chondrocytes (Fig. 5e) as well as elevated levels of the senescence markers p53 and p16 (Fig. 5d). *PTEN*-deficient articular chondrocytes also exhibited a senescence-associated secretory phenotype (SASP), as indicated by elevated expression of *Mmp13* and *Mmp9* (Fig. 5c, f). These results suggest that sustained Akt signaling leads to increased oxidative stress and senescence in articular chondrocytes, which might be responsible for OA pathogenesis.

### Antioxidant therapy relieves chondrocyte senescence and mitigates OA phenotypes in *PTEN*^*fl/fl*^ mice

**Fig. 6:**
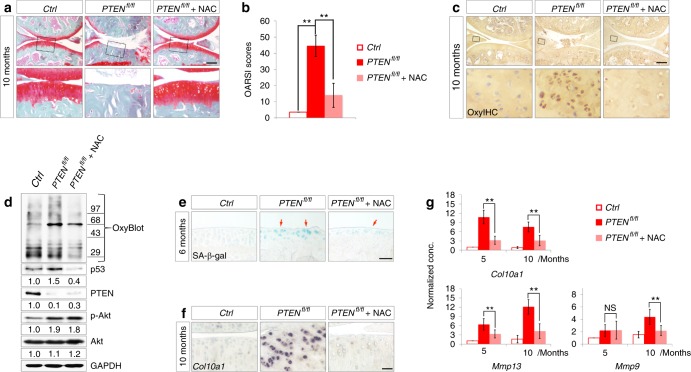
NAC treatment relieves oxidative stress-induced chondrocyte senescence and mitigates OA phenotypes in *PTEN*-deficient mice. **a** Representative images of safranin O staining of articular sections of knee joints from 10-month-old *Ctrl* and *PTEN*^*fl/fl*^ mice as well as *PTEN*^*fl/fl*^ littermates treated with NAC for 9 months (*n* = 12 per group). **b** Quantified pathological changes of each group shown in (**a**). Each value represents the mean ± SEM (*n* = 12 per group). **P *<* 0.01. **c** Representative images of OxyIHC staining of knee joints from each group shown in (**a**) (*n* = 3 per group). The framed area in each picture is shown below at a higher magnification. **d** OxyBlot analysis for protein oxidation and western blot analyses for the levels of p53, PTEN, p-Akt, and Akt in articular cartilage from 6-month-old *Ctrl* and *PTEN*^*fl/fl*^ mice as well as *PTEN*^*fl/fl*^ littermates treated with NAC for 5 months. Quantitative densitometry results are shown below. The GAPDH protein serves as an endogenous normalizer. **e** Representative images of SA-β-gal staining of articular cartilage from 6-month-old *Ctrl* and *PTEN*^*fl/fl*^ mice as well as *PTEN*^*fl/fl*^ littermates treated with NAC for 5 months (*n* = 3 per group). Arrows denote senescent articular chondrocytes. **f** Representative images of *Col10a1* in situ hybridization analysis of knee joints from each group shown in (**a**) (*n* = 3 per group). **g** Representative real-time PCR analyses of *Col10a1*, *Mmp13*, and *Mmp9* expression in articular chondrocytes from 1-, 5- and 10-month-old knee joints. NAC was administered every other day from 1 month to the time of sacrifice. Each value represents the mean ± SEM (*n* = 3 per group). **P *<* 0.01. Scale bars: 250 µm in (**a**) and (**c**), 50 µm in (**e**) and (**f**)

N-acetylcysteine (NAC) is a classic antioxidant that is well known for its ability to minimize intracellular oxidative stress.^30^ Here, we employed long-term intragastric administration of NAC as an antioxidant therapy for the protection of *PTEN*^*fl/fl*^ chondrocytes from intracellular ROS. Ten milligrams of NAC in 0.9% NaCl solution was administered at 1 month, once every other day until sacrifice. Long-term NAC administration at this dose did not affect viability but slightly reduced the body weight of the mice (data not shown). Histological analysis and OA scoring results showed that NAC treatment effectively reduced articular cartilage degeneration in 10-month-old *PTEN*^*fl/fl*^ mice compared with that in their *PTEN*^*fl/fl*^ littermates treated with 0.9% NaCl (Fig. 6a, b). OxyIHC and OxyBlot analyses confirmed the remarkable amelioration of oxidative stress by NAC treatment in chondrocytes from *PTEN*^*fl/fl*^ mice (Fig. 6c, d). As a result, chondrocyte senescence was dramatically arrested by NAC treatment, as shown by decreased SA-β-gal staining (Fig. 6e) and downregulated p53 levels in *PTEN*-deficient chondrocytes (Fig. 6d). The expression of the OA marker gene *Col10a1* and the SASP marker genes *Mmp13* and *Mmp9* in the articular cartilage of *PTEN*^*fl/fl*^ mice were also suppressed by NAC treatment (Fig. 6f, g). These results demonstrate that antioxidant therapy diminishes chondrocyte senescence and recovers OA phenotypes in *PTEN*^*fl/fl*^ mice.

## Discussion

Increasing evidence has shown that cellular senescence plays an important role in aging-related OA development.^16^ Deciphering the mechanisms by which chondrocyte senescence is triggered is the key to understanding the pathogenesis of OA. Here, we provide in vivo genetic evidence that inhibition of Akt signaling by PTEN is required for the maintenance of articular cartilage homeostasis. Loss of PTEN in articular chondrocytes leads to sustained Akt signaling and OA via oxidative stress-induced senescence.

Although many studies favor the chondroprotective role of Akt signaling,^10,11,31^ we found that in vivo, sustained Akt signaling may deteriorate the integrity of articular cartilage. Akt can transduce both proanabolic and procatabolic signaling in response to diverse stimuli during cartilage repair.^13^ Akt signaling may also adopt distinct downstream effectors to perform seemingly opposing functions. For example, mTOR supports protein synthesis and extracellular matrix production during cartilage development and maintenance,^32^ while inhibition of mTOR protects cartilage from experimental OA at least in part by supporting autophagy.^33^ In this study, we showed that human OA samples and mouse OA models manifested intensive and persistent activation of Akt during OA progression. We further demonstrated that constitutive or inducible knockout of *PTEN* in articular chondrocytes led to OA development, providing the first in vivo genetic evidence that prolonged Akt signaling *per se* is sufficient to initiate OA and suggesting that intrinsic Akt signaling should be appropriately balanced to maintain cartilage integrity.

Our study reveals a prosenescence function of sustained Akt signaling that hampers the integrity of articular cartilage. Targeted elimination of p16^Ink4a^-positive senescent cells in transgenic mice verified their contribution to age-related pathologies and dysfunctions.^34,35^ The causal role of senescent chondrocytes in posttraumatic OA pathogenesis has also been addressed using a similar strategy.^16^ The prosenescence role of Akt has been suggested in cancer cells and aging *Caenorhabditis elegans*.^36,37^ In the current study, we showed that *PTEN*-deficient articular chondrocytes exhibited high levels of the senescence inducers p16^Ink4a^ and p53, senescence-associated β-galactosidase activity and typical features of a SASP, unveiling a mechanism by which the senescence of articular chondrocytes is induced. We further showed that *Akt1* knockout significantly rescued the OA phenotypes in *PTEN*-deficient mice, suggesting that pharmacological inhibition of Akt signaling may be a feasible therapeutic strategy for treating OA.

We show that consistently activated Akt signaling in articular chondrocytes causes OA by oxidative stress-induced senescence. Previous in vitro studies have revealed a correlation between oxidative stress-accelerated chondrocyte senescence and altered Akt signaling in OA chondrocytes.^18^^,38–40^ In this study, *PTEN*^*fl/fl*^ articular chondrocytes exhibited higher levels of p-mTOR and p-FoxO1/3a, along with elevated oxidative stress and senescence, suggesting a causal role of oxidative stress in OA in *PTEN*-deficient mice. Consistently, systemic application of the antioxidant NAC efficiently relieved articular chondrocytes from oxidative-stress-induced cellular senescence and SASP and largely mitigated the OA phenotypes in *PTEN*^*fl/fl*^ mice. These findings demonstrate that oxidative stress induced by activated Akt signaling causes chondrocyte senescence and OA, suggesting antioxidants as a potential therapeutic strategy for treating OA. In support of this possibility, very recent preclinical work demonstrates that NAC effectively represses oxidative damage and prevents posttraumatic OA.^41^

## Materials and methods

### Human articular cartilage specimens

Discarded human articular cartilage from orthopedic surgery patients was sourced from Chinese PLA General Hospital, which included 3 traumatic knee joint specimens from individuals with no history of arthritic diseases and 6 knee arthroplasty specimens from OA patients (Supplementary Table S1). The Hospital for Special Surgery Knee-Rating Score (HSS) was employed for the grading of affected OA joints before arthroplasties.^42^ Each specimen was divided into 2 parts, for immunohistochemistry or western blot analyses. The specimens were collected by a protocol that adhered to the tenets of the Declaration of Helsinki and was approved by the Ethics Committee of Chinese PLA General Hospital.

### Mouse strains, tamoxifen induction, and NAC treatment

All mouse experimental protocols were designed according to the recommendation of the Beijing Experimental Animal Regulation Board (SYXK/JING/2005/0031). All mice were on the C57BL/6 genetic background and kept under specific pathogen-free conditions.

Genetically engineered mouse strains included transgenic *Col2a1-Cre* and *Col2a1-Cre*^*ERT2*^ mice,^8,25^ conditional *PTEN*^*fl/fl*^, *Akt1*^*fl/fl*^ and *ROSA*^*EYFP*^ mice.^8,43,44^

Experimental OA model was induced by destabilization of the medial meniscus (DMM) surgery performed on 8-week-old mice. Sham-operated mice were used as controls.^24^ Male mice were used throughout this study.

For induction of Cre^ERT2^ activity, tamoxifen (Sigma, T5648, 0.1 mg·g^–1^ body weight/day) was injected intraperitoneally into mice for 5 consecutive days.

For NAC treatment, *Col2a1-Cre*; *PTEN*^*fl/fl*^ mice were randomly distributed into control and treatment groups. Mice in the treatment group were intragastrically administered freshly prepared 10 mg NAC (Sigma, A9165) in drinking water (adjusted to pH 7.0 using NaOH) every other day from 1 month to the time of sacrifice. Intragastric administration of 0.9% NaCl was applied to the control group in the same way.

### μCT and 3D reconstruction

Mouse hind limbs were scanned by a high-resolution (5 μm cubic voxel size) μCT scanner (Skyscan 1172). 3D model visualization software (CTvox) was used to reconstruct the appearance of the tibial plateau. To allow visual characterization of the density distribution, 3D reconstruction images were color-coded based on Hounsfield Unit (HU) values, which refer to the respective grayscale values.

### Histology, immunohistochemistry, in situ hybridization, OxyIHC, and SA-β-gal staining

For human articular cartilage, the specimens were fixed in 4% paraformaldehyde (PFA) at 4 °C for 2 days, decalcified in 0.5 mol·L^–1^ EDTA in PBS for 2–3 weeks, dehydrated and embedded in paraffin. For mouse articular cartilage, whole knee joints with the tibia and femur were fixed in 4% PFA at 4 °C overnight, decalcified in 0.5 mol·L^–1^ EDTA in PBS for 4–14 days, dehydrated and embedded in paraffin. The paraffin blocks were sectioned at 6 μm thickness.

For histopathologic scoring of mouse OA, a modified form of the OA Research Society International (OARSI) grading system was employed.^45^ Briefly, serial sagittal sections of the medial joint at 30 μm intervals were obtained since the appearance of the inner margin of the medial meniscus, which yielded 10 slides per joint. Safranin O staining was then performed as described previously.^8^ Articular cartilage degradation and subchondral bone changes were assessed in the medial tibial plateau by two blinded observers using an arbitrary grading scale of 0–6 for the severity of cartilage destruction and 0–3 for subchondral bone.^45^ The total grades of 10 sections per joint were then summed to determine the OA score (maximum score of 90). Additional histopathologic grading methods were employed to evaluate synovial changes as well as subchondral bone thickening. The severity of synovial hyperplasia was scored on a grading scale of 0–3 for cell thickness (0 = 1 cells thick, 1 = 2–3 cells thick, 2 = 4–5 cells thick, and 3 = more than 6 cells thick).^46^ The thickness of the subchondral bone plate, i.e., the distance between the osteochondral junction and the marrow space below the tibial plateau, was quantified in ImageJ software.^47,48^ Osteophytosis was identified by safranin O staining of coronal sections of mouse knee joints and was graded by osteophyte size and osteophyte maturity. Briefly, osteophyte size was quantified by a grading scale of 0–3 (0 = no osteophytes, 1 = thickness similar to the adjacent cartilage, 2 = 1–3 times the thickness of the adjacent cartilage, and 3 = more than 3 times the thickness of the adjacent cartilage). Osteophyte maturity was assessed by a grading scale of 0–3 (0 = no osteophytes, 1 = predominant cartilage, 2 = mixed cartilage and bone with vascular invasion and endochondral ossification, and 3 = predominant bone).^49^

For immunohistochemistry, sections were rehydrated and subjected to antigen retrieval. The retrieval protocol for detecting GFP and phospho-Akt is as follows: slides were digested in 20 µg·mL^–1^ proteinase K at 37 °C for 10 min, followed by incubation in 10 mmol·L^–1^ citrate buffer (pH 6.0) at 50 °C for 16 h. The primary antibodies were monoclonal rabbit anti-GFP (Cell Signaling, 2 956, 1:200) and monoclonal rabbit anti-phospho-Akt (Ser473) (Cell Signaling, 4 060, 1:200). The signal was visualized with a Polink-1 HRP Kit for Rabbit (GBI Labs, D13–110) with DAB as the chromogen. Negative controls were performed using nonimmunized IgG to replace the primary antibody (Fig. 1a and Supplementary Fig. S5a).

For OxyIHC, the knee joints were fixed in Methacarn fixative solution (10% glacial acetic acid, 30% trichloromethane, 60% methanol) at 4 °C overnight and decalcified in 0.5 mol·L^–1^ EDTA (without PFA) in PBS. Paraffin sections (6 μm) were used according to the Millipore OxyIHC^TM^ oxidative stress detection kit protocol (Millipore, S7450). Negative controls were performed with the Derivatization Control Solution from the Millipore OxyIHC^TM^ oxidative stress detection kit (Supplementary Fig. S5b).

For in situ hybridization, DIG-labeled antisense probes were generated from linearized template vectors with a DIG RNA Labeling Kit (Roche Life Science, 11277073910). In situ hybridization was performed using standard procedures.^50^ The signal was visualized with an AP-conjugated sheep-anti-DIG antibody (Roche Life Science, 11093274910, 1:2 000) with BCIP/NBT as the chromogen. Negative controls were performed using sense probes (Supplementary Fig. S5c and S5d).

For SA-β-gal staining, 10 μm cryosections of tibial articular cartilage were stained with the dye solution (1 mg·mL^–1^ X-Gal, 0.12 mmol·L^–1^ K_3_Fe[CN]_6_, 0.12 mmol·L^–1^ K_3_Fe[CN]_6_·3H_2_O, 1 mmol·L^–1^ MgCl_2_, pH 6.0) for 6 h–12 h according to the reported protocol.^51^

### Western blot and OxyBlot analysis

Human articular cartilage specimens were ground in liquid nitrogen. The cartilaginous surface of dissected mouse knee joints was sliced by a fine knife under a stereomicroscope, collected and ground in liquid nitrogen. The sample for each genotype was obtained from 3–10 mice. Primary antibodies against PTEN (Cell Signaling, 9 188, 1:1 000), phospho-Akt (Ser473) (Cell Signaling, 4 060, 1:1 000), Akt (pan) (Cell Signaling, 4 685, 1:1 000), phospho-mTOR (Ser2448) (Cell Signaling, 5 536, 1:1 000), phospho-FoxO1 (Thr24)/FoxO3a (Thr32) (Cell Signaling, 9 464, 1:1 000), FoxO1 (Cell Signaling, 2 880, 1:1 000), p53 (Cell Signaling, 2 527, 1:500), p16 (Abcam, ab108349, 1:500) and GAPDH (Sigma-Aldrich, G8795, 1:5 000) were employed for Western blot analyses. OxyBlot was operated according to the Millipore OxyBlot^TM^ protein oxidation detection kit protocol (Millipore, S7150). Western blot results were quantified by densitometry using the ImageJ program. The value for each protein was normalized by its corresponding GAPDH level. The normalized protein level in each control or sham group was then set as 1.0 (100%), and the littermates were presented as the ratio to the corresponding control or sham group.

### Real-time PCR analyses

Ground articular cartilage obtained from 3–10 mice of each genotype was extracted using TRIZOL (Life Technologies, 15596-026). Total RNA was then reverse-transcribed using the mRNA Selective PCR Kit (Takara, RR025A). Real-time PCR was performed with the Roche LightCycler 2.0 system using a SYBR Select Master Mix (Life Technologies, 4472908) with four repeats for each sample. Expression values were normalized to *Hprt*.

### Statistical analysis

The results are presented as the mean ± SEM. Quantitative data were tested for normality and homogeneity of variance. For comparisons between two groups, an unpaired Student’s *t*-test was used. For multiple comparisons among more than two groups, data were analyzed by one-way analysis of variance (ANOVA), followed by Dunnett’s test. Semiquantitative OA indices were analyzed using one-way ANOVA and the Kruskal–Wallis test, followed by Dunn’s multiple comparison post hoc test. Differences were considered significant when the *P*-value was less than 0.05.

## Supplementary information

Supplementary figures and tables
