# Supplementary material for: Sustained Akt signaling in articular chondrocytes causes osteoarthritis via oxidative stress-induced senescence in mice
Source: Bone Res. 2019 Aug 5;7:23. doi: 10.1038/s41413-019-0062-y (PMC6804644; doi:10.1038/s41413-019-0062-y)
Supplement: Supplementary file 1 — Supplementary figures and tables [file 41413_2019_62_MOESM1_ESM.pdf]

Table S1. Summarized information on human cartilage specimens.

OA cartilage samples from knee arthroplasties

ID NO.	Gender	Age (year)	Disease duration* (year)	HSS scores	Medications
1	F	65	15	43 (L)	NSAIDs; HA
2	M	59	6	59 (R)	NSAIDs
3	F	66	10	57 (L)	NSAIDs; HA
4	F	66	3	30 (L)	NSAIDs
5	F	69	2	41 (R)	NSAIDs
6	F	70	22	53 (R)	NSAIDs

Cartilage samples from traumatic knee joints

ID NO.	Gender	Age (year)	Causes of trauma	OA history
1	F	50	running sprain	No
2	F	54	staircase fall	No
3	M	50	car accident	No

*Since diagnosis; F: female, M: male; L: left, R: right; NSAIDs: nonsteroidal anti-inflammatory drugs, HA: hyaluronic acid.

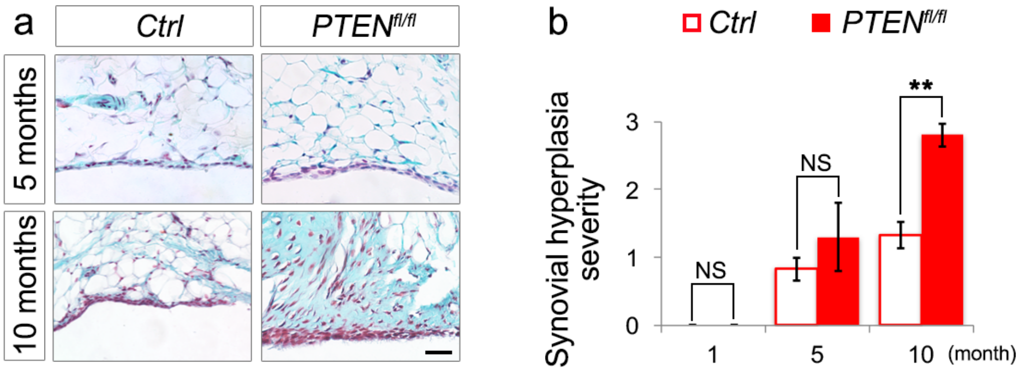

Fig. S1. Synovial hyperplasia occurs in *PTEN*-deficient mice. **a** Representative images of safranin O staining of synovia from *Ctrl* and *PTEN*$^{fl/fl}$ mice at 5 and 10 months (n = 6 per group). **b** Quantified synovial changes from *Ctrl* and *PTEN*$^{fl/fl}$ mice at 1, 5 and 10 months. Each value represents Mean ± SEM (n = 6 per group). NS: not significant; $^{**}p < 0.01$. Scale bar: 50 μm.

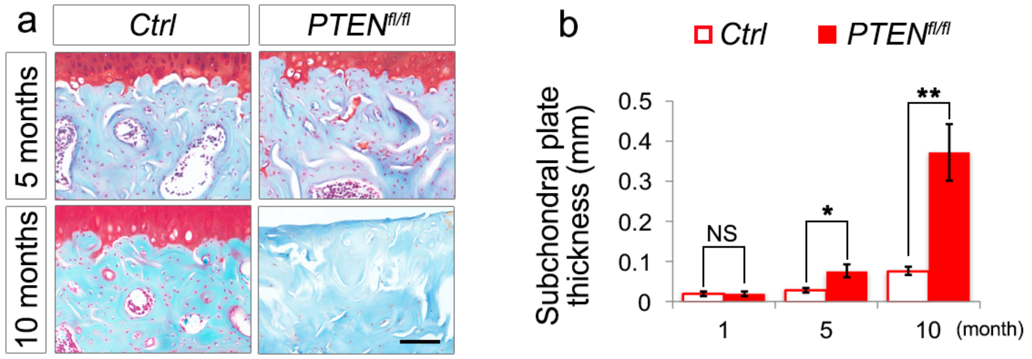

Fig. S2. Subchondral bone changes in *PTEN*-deficient mice. **a** Representative images of safranin O staining of subchondral bones from *Ctrl* and $PTEN^{fl/fl}$ mice at 5 and 10 months (n = 6 per group). **b** Quantified subchondral plate thickening in *Ctrl* and $PTEN^{fl/fl}$ mice at 1, 5 and 10 months. Each value represents Mean ± SEM (n = 6 per group). NS, not significant; *$p < 0.05$; **$p < 0.01$. Scale bar: 100 μm.

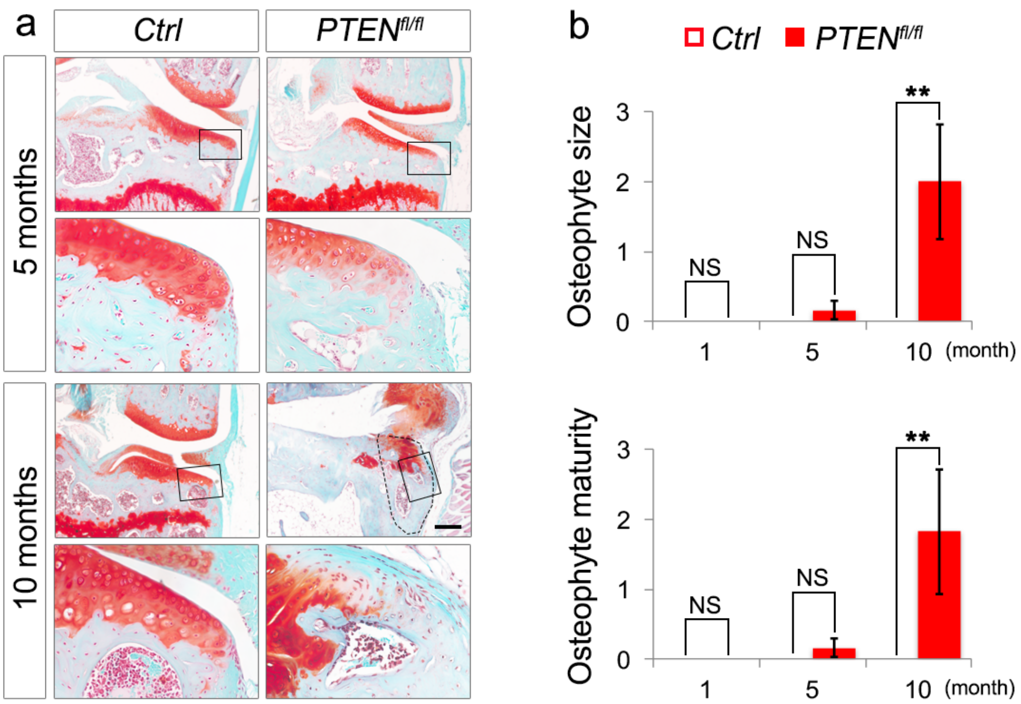

Fig. S3. Osteophytosis occurs in *PTEN*-deficient mice. **a** Representative images of safranin O staining of coronal sections from *Ctrl* and *PTEN*$^{fl/fl}$ mice at 5 and 10 months (n = 6 per group). The framed area in each picture is shown below at a higher magnification. **b** The scoring results of osteophyte size and osteophyte maturity in *Ctrl* and *PTEN*$^{fl/fl}$ mice at 1, 5 and 10 months. Each value represents Mean ± SEM (n = 6 per group). NS, not significant; $**p < 0.01$. Scale bar: 250 μm.

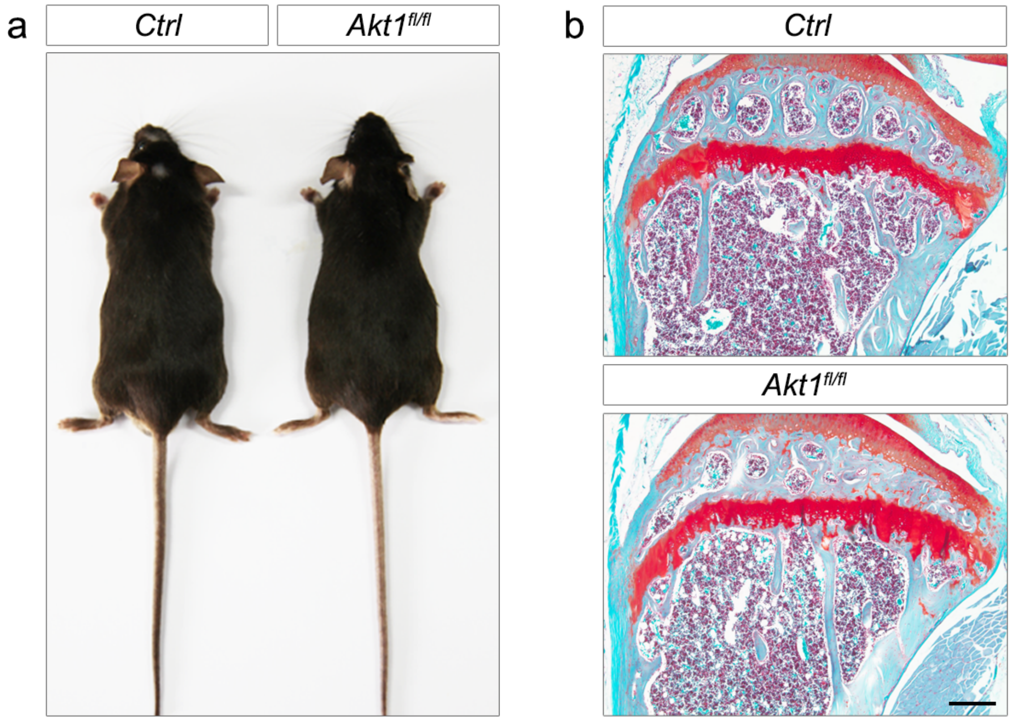

Fig. S4. *Akt1*-deficiency in chondrocytes does not cause visible defects in skeleton development. **a** Gross appearance of 6-month-old *Ctrl* and *Akt1*$^{fl/fl}$ mice. **b** Representative images of safranin O staining of tibial growth plates from 6-month-old *Ctrl* and *Akt1*$^{fl/fl}$ mice (n = 6 per group). Scale bar: 250 μm.

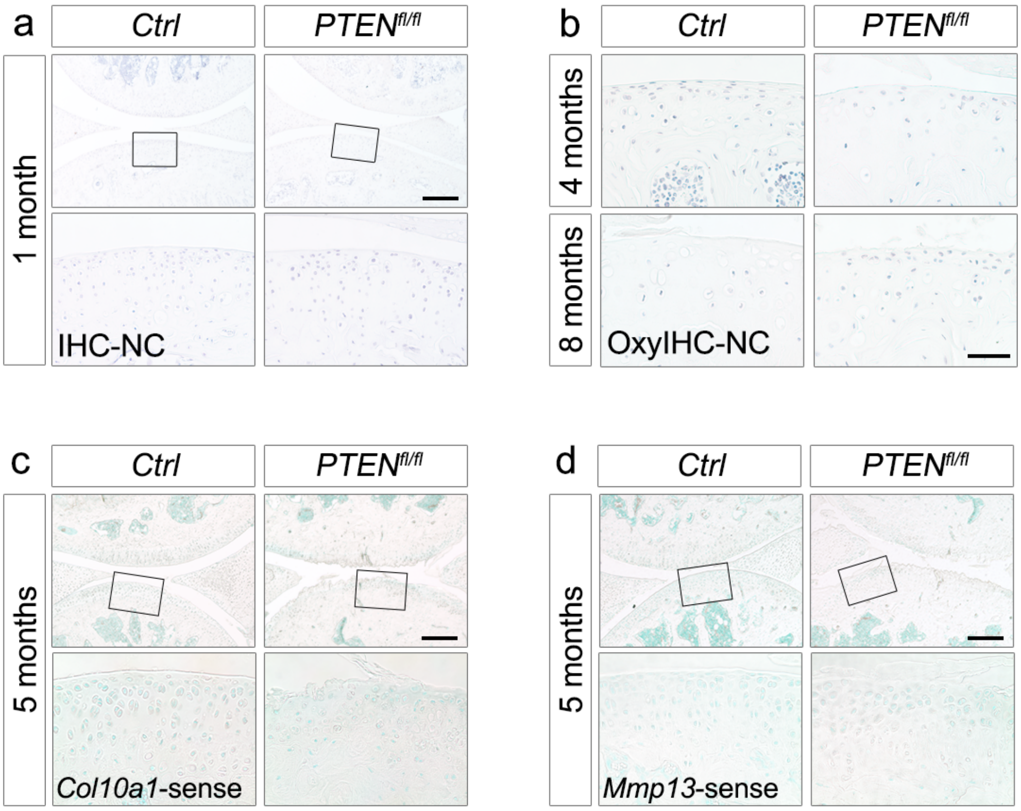

Fig. S5. Negative controls for chromogen-based histological staining methods. **a** Representative images of immunohistochemical staining performed on sections of 1-month-old mouse knee joints using non-immunized rabbit IgG as the negative control (NC). The framed area in each picture is shown below at a higher magnification. **b** Representative images of OxyIHC performed on sections of 4- and 8-month-old mouse knee joints. Negative control reactions were performed with the Derivatization Control Solution. **c, d** Representative images of *in situ* hybridization analyses of 5-month-old mouse knee joints using *Col10a1* and *Mmp13* sense probes, respectively. The framed area in each picture is shown below at a higher magnification. Scale bars: 50 μm in **b**, 200 μm in **a, c** and **d**.
